# EasyAmplicon 2: Expanding PacBio and Nanopore Long Amplicon Sequencing Analysis Pipeline for Microbiome

**DOI:** 10.1002/advs.202512447

**Published:** 2025-10-27

**Authors:** Hao Luo, Defeng Bai, Zhihao Zhu, Salsabeel Yousuf, Haifei Yang, Jiani Xun, Meiyin Zeng, Yao Wang, Yunyun Gao, Kai Peng, Shanshan Xu, Yuanping Zhou, Tianyuan Zhang, Chuang Ma, Huiyu Hou, Xiulin Wan, Yang Zhou, Baolei Jia, Shi Huang, Renyou Gan, Tao Wen, Tong Chen, Xia Chen, Xiaofang Li, Yong‐Xin Liu

**Affiliations:** ^1^ Genome Analysis Laboratory of the Ministry of Agriculture and Rural Affairs Agricultural Genomics Institute at Shenzhen Chinese Academy of Agricultural Sciences Shenzhen 518120 China; ^2^ Zhanjiang Key Laboratory of Human Microecology and Clinical Translation Research, School of Basic Medical Sciences Guangdong Medical University Zhanjiang 524023 China; ^3^ College of Life Sciences Qingdao Agricultural University Qingdao 266109 China; ^4^ School of Ecology and Nature Conservation Beijing Forestry University Beijing 100083 China; ^5^ Jiangsu Co‐Innovation Center for Prevention and Control of Important Animal Infectious Diseases and Zoonoses College of Veterinary Medicine Yangzhou University Yangzhou Jiangsu 225000 China; ^6^ School of Food and Biological Engineering Hefei University of Technology No.193 Tunxi Road Hefei Anhui 230009 China; ^7^ School of Horticulture Anhui Agricultural University Hefei 230000 China; ^8^ Oil Crops Research Institute Chinese Academy of Agricultural Sciences Wuhan 430062 China; ^9^ Xianghu Laboratory Hangzhou 310000 China; ^10^ Faculty of Dentistry The University of Hong Kong Hong Kong SAR 999077 China; ^11^ Department of Food Science and Nutrition Faculty of Science The Hong Kong Polytechnic University Hong Kong Kowloon 999077 China; ^12^ Jiangsu Provincial Key Lab for Solid Organic Waste Utilization Key Lab of Organic‐Based Fertilizers of China Jiangsu Collaborative Innovation Center for Solid Organic Wastes Educational Ministry Engineering Center of Resource‐Saving Fertilizers Nanjing Agricultural University Nanjing Jiangsu 210000 China; ^13^ State Key Laboratory for Quality Assurance and Sustainable Use of Dao‐di Herbs National Resource Center for Chinese Materia Medica China Academy of Chinese Medical Sciences Beijing 100091 China; ^14^ Ningbo Key Laboratory of Human Microbiome and Precision Medicine Central Laboratory of the Medical Research Center The First Affiliated Hospital of Ningbo University Ningbo 315000 China; ^15^ Center for Agricultural Resources Research Institute of Genetics and Developmental Biology Chinese Academy of Sciences Shijiazhuang 050021 China

**Keywords:** amplicon, microbiome, Nanopore, PacBio, Pipeline

## Abstract

In the past decade, third‐generation sequencing technologies (such as PacBio (Pacific Biosciences) and Nanopore) have become gradually matured and are widely used for microbial taxonomy and quantification. Compared with Illumina sequencing, PacBio or Nanopore has advantages with long reads and high resolution in taxonomic classification. However, there is currently a lack of an easy‐to‐use, reproducible, and community‐supported pipeline for PacBio or Nanopore amplicon sequencing data analysis. To address this shortcoming, the highly cited EasyAmplicon is updated to version 2, a pipeline fully supporting third‐generation full‐length amplicon data. EasyAmplicon 2 is a user‐friendly pipeline that embraces data analysis and visualization options for data obtained from various sequencing technologies (Illumina, BGI (Beijing Genomics Institution), PacBio, Nanopore or Qitan). It integrates popular tools such as DADA2 and Emu, and provides a workflow from raw data to publication‐ready visualizations. EasyAmplicon 2 inherits the advantages of the previous version and further optimizes the visualization part. The updated version of the pipeline includes data preprocessing, annotation, and quantification of amplicon sequence variants, intergroup comparison, and visualization for third‐generation sequencing. EasyAmplicon 2 provides a simple and easy‐to‐use analysis environment for long‐read amplicon sequencing data analysis. It is available for free on GitHub (https://github.com/YongxinLiu/EasyAmplicon).

## Graphical abstract

EasyAmplicon 2 is an updated, user‐friendly, and community‐supported pipeline designed for full‐length amplicon data from long‐read sequencing technologies such as PacBio and Nanopore. It supports multiple platforms (Illumina, PacBio, Nanopore, etc.), integrates tools like DADA2 and Emu, and offers a complete workflow from raw data processing to high‐quality visualizations. EasyAmplicon 2 enhances visualization features and provides reproducible analysis for microbial annotation and quantification. It is freely available on GitHub: https://github.com/YongxinLiu/EasyAmplicon.

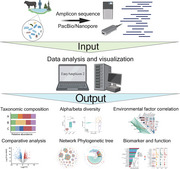

## 1 Introduction

Amplicon sequencing has long been a cornerstone of microbial community profiling, enabling researchers to characterize the taxonomic and ecological diversity of microbiomes across diverse environments, including human,^[^ ^1^, ^2^ ^]^ animals,^[^ ^3^ ^]^ plants,^[^ ^4^ ^]^ and a wide range of ecosystems.^[^ ^5^, ^6^, ^7^ ^]^ For over a decade, traditional short‐read sequencing platforms like Illumina have dominated the field, offering reliable yet often fragmented insights into microbial diversity through targeted amplification of partial regions within conserved marker genes (e.g., 16S/18S rRNA, ITS).^[^ ^8^, ^9^, ^10^ ^]^ However, the limited resolution of short‐read amplicons typically covering only hyper‐variable subregions poses a significant barrier to get accurate species‐level classification and robust phylogenetic reconstruction.^[^ ^11^ ^]^

With the advent of long‐read sequencing technologies, such as Pacific Biosciences (PacBio) and Oxford Nanopore Technologies (ONT),^[^ ^12^, ^13^, ^14^ ^]^ researchers are now able to obtain full‐length amplicon sequences that span entire genes, including the ≈1.5 kb bacterial 16S rRNA gene or multi‐locus targets.^[^ ^11^ ^]^ These long‐read sequences provide unprecedented taxonomic resolution, facilitating species‐level classification, more accurate phylogenetic reconstruction, and enhanced functional interpretation of microbial communities.^[^ ^8^, ^15^, ^16^ ^]^ Although long‐read sequencing initially exhibited relatively high per‐read error rates during the early stages of its development, recent advancements have improved sequence accuracy to over 97%.^[^ ^13^, ^17^, ^18^ ^]^ With continued optimization and development of third‐generation sequencing technology, limitations such as high per‐read error rates are expected to be progressively overcome. The era of widespread application of long‐read sequencing is approaching. However, the analysis of full‐length amplicon data poses distinct challenges, including limited compatibility with conventional short‐read analysis pipelines and a lack of standardized, end‐to‐end workflows for processing raw data into high‐quality, publication‐ready outputs.

To bridge this gap, we present EasyAmplicon 2, a streamlined, modular, and open‐source pipeline specifically designed for full‐length amplicon sequencing. EasyAmplicon 2 is an updated and enhanced version of EasyAmplicon pipeline 1.0.^[^ ^19^, ^20^ ^]^ The version 1.0 focuses on the analysis of short‐read amplicon data, and integrates commonly used amplicon processing tools: VSEARCH^[^ ^21^ ^]^ for reads merging, primer trimming, quality control, dereplication, and feature table generation; USEARCH^[^ ^22^, ^23^ ^]^ for OTU (Operational taxonomic unit)/ASV (Amplicon sequence variant) clustering, diversity analysis, and getting quantitative feature tables; QIIME 2^[^ ^24^ ^]^ for short‐read amplicon processing; PICRUSt2^[^ ^25^ ^]^ and FAPROTAX^[^ ^26^ ^]^ for functional prediction/annotation; and BugBase^[^ ^27^ ^]^ for bacterial phenotype prediction. Compared with version 1.0, EasyAmplicon 2 integrates an R framework with a Snakemake‐based automated workflow in Linux, enabling reproducible processing from raw data to feature table analysis and visualization. It supports PacBio and ONT datasets, incorporating quality control, denoising, chimera removal, and taxonomic assignment. EasyAmplicon 2 also provides multiple reference databases (SILVA,^[^ ^28^ ^]^ GTDB,^[^ ^29^ ^]^ NCBI,^[^ ^30^ ^]^ Emu default database,^[^ ^11^ ^]^ UNITE,^[^ ^31^ ^]^ Greengenes,^[^ ^32^ ^]^ and RDP^[^ ^33^ ^]^) and compatible versions of tools (Cutadapt,^[^ ^34^ ^]^ DADA2,^[^ ^35^ ^]^ Emu,^[^ ^11^ ^]^ USEARCH,^[^ ^22^, ^23^ ^]^ and VSEARCH^[^ ^21^ ^]^), enabling high‐resolution taxonomic profiling with minimal manual effort. While EasyAmplicon 2 primarily focuses on bacterial annotation and analysis, we recognize recent advances in viral analysis pipelines based on long‐read sequencing, such as INSaFLU‐TELEVIR^[^ ^36^ ^]^ and VirDetector.^[^ ^37^ ^]^ A workflow for viral annotation using long‐read sequencing data is still under development and will be released in the future. Meanwhile, in order to address the limitations (e.g., lack of long‐read data analysis, slow upload speed, long waiting/running time, few adjustable parameters, and lack of customizability) of online platforms (e.g., Qiita,^[^ ^38^ ^]^ MGnify,^[^ ^39^ ^]^ gcMeta,^[^ ^40^ ^]^ and LotuS2^[^ ^41^ ^]^), EasyAmplicon 2 is developed as a code‐based platform that facilitates reproducible analysis and enables personalized modification through customizable scripts, as with other user‐friendly pipeline or platforms we have developed, such as previous released EasyAmplicon,^[^ ^19^, ^20^ ^]^ EasyMetagenome,^[^ ^42^ ^]^ MicrobiomeStatPlots,^[^ ^43^ ^]^ and EasyNanoMeta.^[^ ^44^ ^]^

Compared to existing tools and pipelines, which focus primarily on short‐read data or demand complex setups for long‐read data processing,^[^ ^45^, ^46^ ^]^ EasyAmplicon 2 prioritizes accessibility, reproducibility, and flexibility. The pipeline provides both command‐line and interactive RStudio interfaces, supports batch processing of multiple samples or sequencing regions, and offers over 30 customizable visualization styles suitable for ecological interpretation and publication‐ready figures. EasyAmplicon 2 addresses the specific demands of long‐read amplicon data, filling a critical gap in microbiome bioinformatics. It empowers researchers to unlock the full potential of full‐length sequencing technologies across microbial ecology, clinical microbiology, agriculture, and beyond.

## 2 Result

### 2.1 Overview of EasyAmplicon 2

EasyAmplicon 2 is a modular yet fully integrated pipeline for analyzing full‐length amplicon data and generating publication‐ready visualizations, optimized for long‐read sequencing technologies like PacBio and ONT. It is designed for efficient execution on both personal laptops and high‐performance computing (HPC) servers, incorporating several new software into the pipeline (**Table** **1**), and generating feature tables and publication‐ready figures to facilitate in‐depth interpretations. For full‐length 16S rRNA samples (PacBio HiFi reads, ≈20000 reads/sample), EasyAmplicon 2 completes end‐to‐end analysis (quality control, denoising, taxonomic assignment, and visualization) less than 2 h using a dual‐core processor (2.3 GHz) with under 6 GB RAM. This high efficiency of EasyAmplicon 2 enables seamless scaling from small sample exploratory studies to large‐cohort projects.

**Table 1:** New software and packages included in EasyAmplicon 2.

Software	Function in the pipeline	Website
minimap2	A versatile sequence alignment program that aligns DNA or mRNA sequences against a large reference database.	https://github.com/lh3/minimap2
samtools	Tools for manipulating next‐generation sequencing data.	https://github.com/samtools/samtools
cutadapt	Finds and removes adapter sequences, primers, poly‐A tails and other types of unwanted sequence from your high‐throughput sequencing reads.	https://github.com/marcelm/cutadapt
DADA2	Accurate sample inference from amplicon data with single nucleotide resolution.	https://github.com/benjjneb/dada2
emu	Species‐level taxonomic abundance quantification for full‐length 16S reads	https://github.com/treangenlab/emu
amplicon	R package "amplicon" for amplicon data statistics and visualization in microbiome.	https://github.com/microbiota/amplicon
VennDiagram	R package for generating hig‐resolution venn and euler plots.	https://cran.r‐project.org/web/packages/VennDiagram/index.html
pheatmap	R package for plotting heatmaps.	https://cran.r‐project.org/web/packages/pheatmap/index.html
microeco	R package for microbial cummunity ecology data analysis.	https://cran.r‐project.org/web/packages/microeco/index.html
DESeq2	Differential expression of RNA‐seq data using the Negative Binomial.	https://github.com/thelovelab/DESeq2
ComplexHeatmap	R package provides a highly flexible way to arrange multiple heatmaps and supports various annotation graphics.	https://github.com/jokergoo/ComplexHeatmap
linkET	R package visualizes simply and directly a matrix heatmap based on “ggplot2”.	https://github.com/Hy4m/linkET
ggtree	R package for visualization and annotation of phylogenetic trees.	https://github.com/YuLab‐SMU/ggtree
ggtreeExra	R package for adding geom layers on circular or other layout tree of “ggtree”.	https://github.com/YuLab‐SMU/ggtreeExtra
ggClusterNet	R package for microbiome network visualization.	https://github.com/taowenmicro/ggClusterNet/
ggraph	Creates layout for tree map and circle packing chart.	https://github.com/thomasp85/ggraph
randomForest	Classification and regression based on a forest of trees using random inputs.	https://cran.r‐project.org/web/packages/randomForest/index.html
circlize	Circular visualization.	https://github.com/jokergoo/circlize

Note: This table is updated based on the previous of EasyAmplicon.^[^ ^19^ ^]^

**Figure 1:**
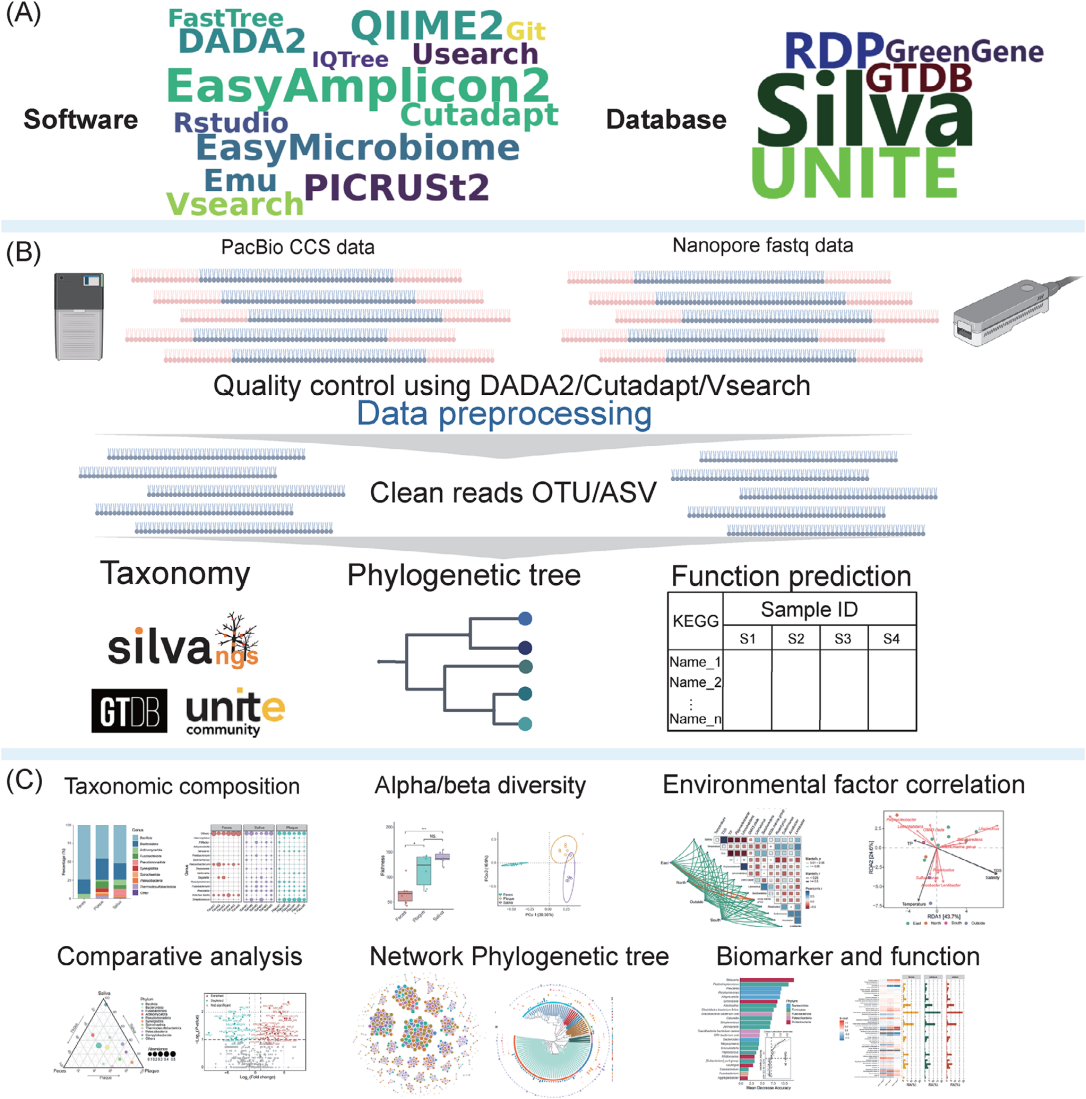
Overview of EasyAmplicon 2 for analyzing PacBio and Nanopore amplicon sequences. A) Software and database installation. B) Core pipeline supporting both PacBio CCS (Circular consensus sequences) and Nanopore FASTQ data processing. C) Downstream statistical analysis and visualizations. Some elements in this Figure 1B are created by BioRender (https://app.biorender.com/).

**Figure 2:**
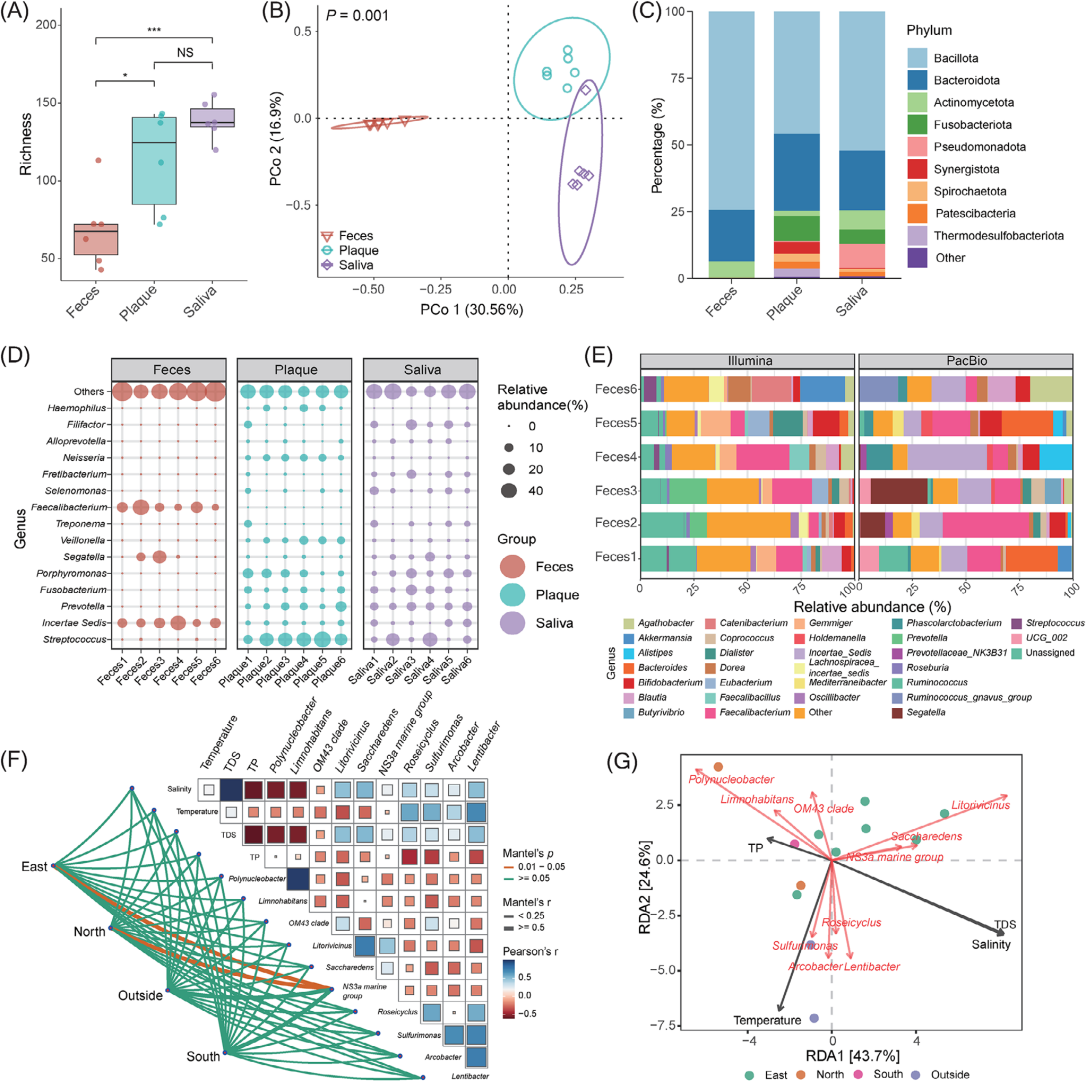
Example plots generated using EasyAmplicon 2. A) The box plot for comparison of alpha diversity between groups. B) Principal coordinate analysis (PCoA) shows the comparison of beta diversity. C) A stacked bar plot shows the change of relative abundance of bacteria at the phylum level of three groups. (D) The grouped bubble plot shows the variation in the relative abundance of microbial genera between samples. E) The faceted stacked bar plot shows the difference in relative abundance of microbial genera between Illumina and PacBio. F) The mantel test correlation heatmap shows the correlation of environmental variables and the relative abundance of key microbial genera. G) Redundancy analysis (RDA) shows the relationship between environmental variables and the relative abundance of key microbial genera. (*n* = 6 samples per condition; ^*^ represents *p* < 0.05; ^***^ represents *p* < 0.001; “NS” represents not significant).

**Figure 3:**
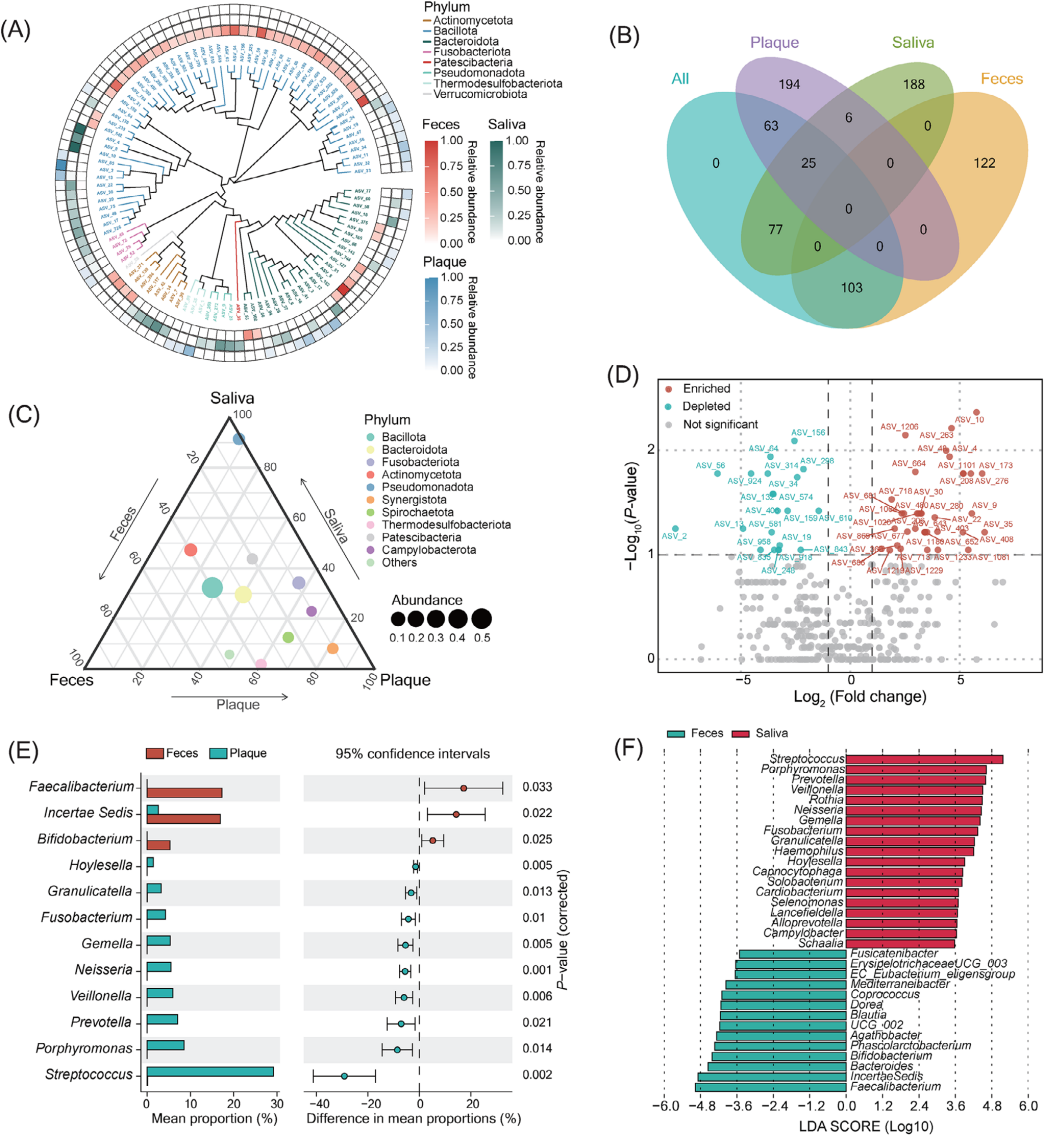
Difference analysis of bacterial data using EasyAmplicon 2. A) The phylogenetic tree shows the relative abundance of amplicon sequence variants (ASVs) in different groups. B) A venn plot shows the number of the same and different core ASVs between groups. C) The ternary plot shows the relative abundance of microbial phyla at the three sampling sites. D) The volcano plot shows the differences (enriched in red and depleted in green) in the relative abundance of ASVs between groups. E) The extended error bar plot shows the relative abundance differences and 95% confidence intervals of microbial genera between feces and plaque groups. F) LEfSe analysis shows differences in the relative abundance of microbial genera between the feces and saliva groups. (*n* = 6 samples per condition; the significance test method used in the volcano and extended bar plot is a two‐sided Wilcoxon rank‐sum test).

EasyAmplicon 2 executes an end‐to‐end workflow from raw data inputs to feature table analysis and publication‐ready visualizations (**Figure** **1**). The pipeline consists of three main modules: 1) installation (Figure 1A), 2) preprocessing and taxonomic assignment (Figure 1B), and 3) visualization (Figure 1C).

### 2.2 Running the Pipeline via Command Line or RStudio

Users can initiate the workflow via the command‐line shell script (Install.sh, pipeline.sh, PacBio_pipeline.sh, Nanopore_pipeline.sh). After specifying the working directory and modifying a few parameters (such as primer sequence or reference database), the entire pipeline can be executed step‐by‐step. Users only need to provide raw sequencing data and sample metadata; all subsequent steps, including quality filtering, error correction, clustering, and visualization, are fully automated by EasyAmplicon 2.

For users preferring command‐line environments, all steps can be executed in Linux/Mac terminals, Windows Git Bash, or remote online servers, offering maximum flexibility across diverse computing platforms. By default, all figures are exported in PDF format, ensuring compatibility with scientific publication standards and allowing for easy downstream editing. The pipeline also integrates commonly used data analysis and visualization codes to facilitate the rapid generation and interpretation of results.

### 2.3 Species Annotation and Microbial Diversity Profiling

EasyAmplicon 2 enables microbial diversity and composition analysis from OTU/ASV tables and taxonomy annotations derived from Illumina, PacBio, or Nanopore sequencing data (**Figure** **2**A–E; Figures S1A–G and S2A–G, Supporting Information). Box plots show that plaque and saliva samples harbor significantly higher alpha diversity (richness) than fecal samples, with no difference between plaque and saliva (Figure 2A; Figure S1A, Supporting Information). No significant richness difference is observed among groups with Nanopore data (Figure S2A, Supporting Information). Principal coordinate analysis (PCoA) reveals significant beta diversity differences among groups (*p* < 0.05; Figure 2B; Figures S1B and S2B, Supporting Information). Phylum‐level stacked bar plots highlight distinct community structures: Bacillota is enriched in feces with Illumina/PacBio data (Figure 2C; **Figure** S1C, Supporting Information), whereas Pseudomonadota dominates the outside group with Nanopore data (Figure S2C, Supporting Information). The donut plot shows the relative abundance composition of the main phyla of these four groups (South, North, East, and Outside) (Figure S2D, Supporting Information). The bubble plot visualizes variations in the relative abundance of microbial genera across individual samples and among different groups (Figure 2D; Figures S1D and S2E, Supporting Information). A faceted stacked bar plot shows clear discrepancies in species composition between short‐read (Illumina) and long‐read (PacBio) sequencing, even from the same samples (Figure 2E). Beyond diversity and composition, Mantel tests and redundancy analysis (RDA) evaluate environmental impacts. Mantel heatmaps quantify correlations between salinity, temperature, TDS, TP, and bacterial genera (Figure 2F). RDA visualizes how these gradients shape community distribution across regions, with arrows indicating the direction and strength of environmental and microbial drivers (Figure 2G).

### 2.4 Differential Abundance Analysis Across Sample Groups

In addition to diversity and environmental analyses, EasyAmplicon 2 provides advanced visualization tools for intergroup comparisons (**Figure** **3**A–F). The phylogenetic tree (without evolutionary distances) displays ASV relative abundance across groups (feces: red, plaque: blue, saliva: green; **Figure** 3A). A Venn plot shows shared and unique amplicon sequence variants (ASVs) among groups, with Evenn^[^ ^47^ ^]^ recommended for customizable diagrams (Figure 3B). Chord diagrams highlight genus‐level differences across Illumina and Nanopore datasets (Figures S1E and S2E, Supporting Information). Ternary plots illustrate phylum‐level distributions, where Bacillota dominates feces, plaque, and saliva (Figure 3C; Figure S1F, Supporting Information), while Pseudomonadota is enriched in Nanopore samples (Figure S2G, Supporting Information). The volcano plot reveals differentially abundant ASVs, with ASV_10 significantly enriched in feces (Figure 3D). The extended error bar plot quantified genus‐level differences with 95% confidence intervals (Figure 3E; Figure S1G, Supporting Information). Alternatively, researchers can perform the same analysis using the STAMP (Statistical analysis of metagenomic profiles) software.^[^ ^48^ ^]^ Finally, the LEfSe (Linear discriminant analysis effect size) analysis via Wekemo Biocloud^[^ ^49^ ^]^ found *Streptococcus* was the most significantly enriched genus in the saliva group, while *Faecalibacterium* was most significantly enriched in the feces group (Figure 3F).

### 2.5 Functional Prediction and Biomarker Identification

**Figure 4:**
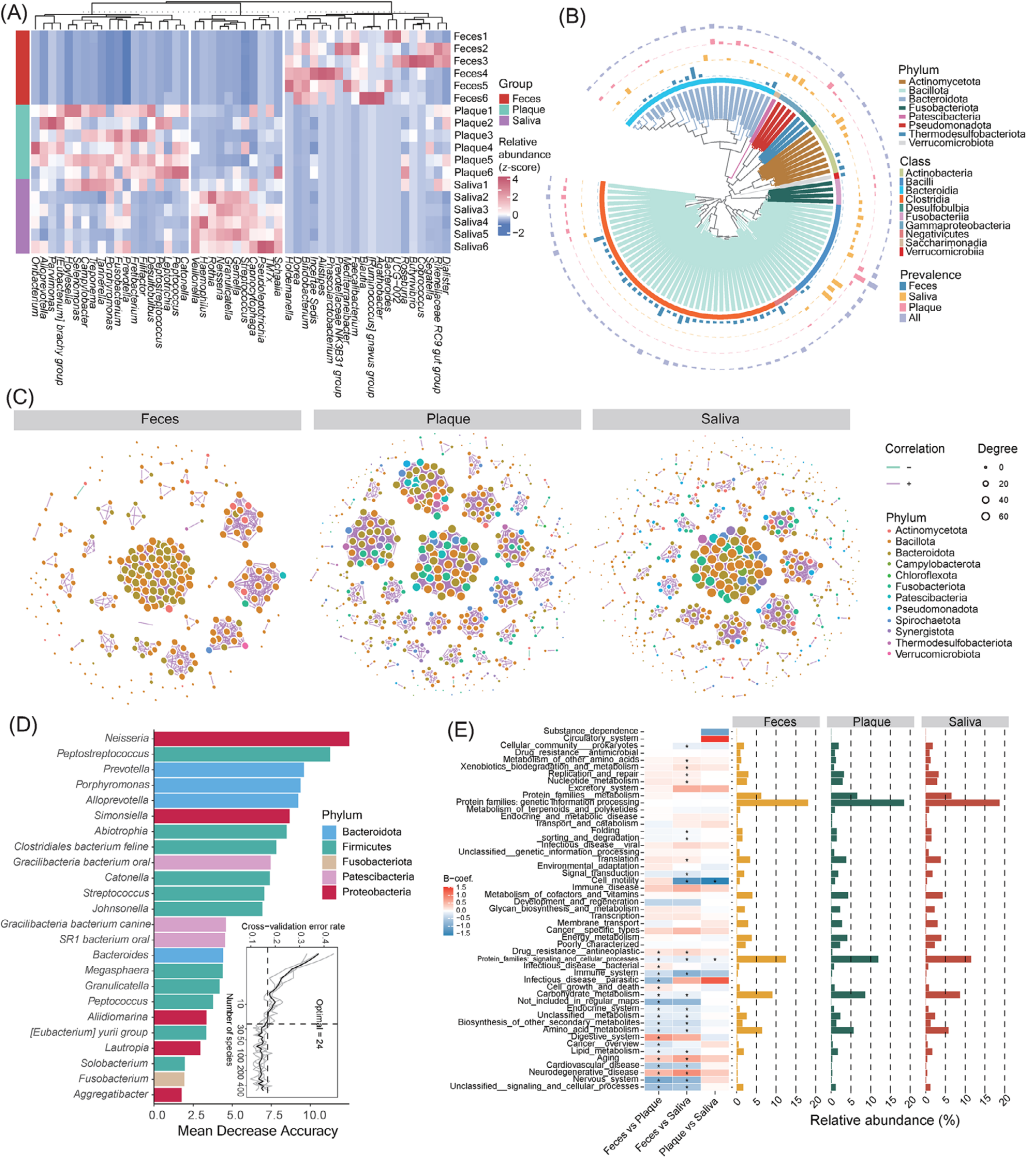
Advanced analysis of bacteria long‐read amplicon using EasyAmplicon 2. A) The heatmap shows the relative abundance of the top 50 microbial genera in different samples. B) Phylogenetic tree with evolutionary distances showing the prevalence of different amplicon sequence variants (ASVs) in different samples. The colored branches inside represent the different phylum. The outer color bands show the different class. The four bar plots in the outer circle show the prevalence of different ASVs in feces (blue), saliva (orange), plaque (pink), and all samples (feces, saliva and plaque combined) (purple). C) The co‐occurrence network shows the differences in ASV interactions between different groups. The different colors of the nodes represent different phylum. The colored edges represent the positive (purple) and negative (green) interactions. D) Biomarkers obtained by random forest model using data from PacBio long‐read sequencing. The bar represents the importance of biomarkers. The internal error rate line plot shows the basis for selecting the optimal number of biomarkers. E) The heatmap combined with the bar plot shows the difference in relative abundance of functions between different groups. In the left heatmap, red indicates increase and blue indicates decrease. The bar plots on the right show the relative abundance of different functions in different groups. (*n* = 6 samples per condition; the significance test method used in the heatmap combined with the bar plot is MaAsLin2, ^*^ represents *p* < 0.05).

We further enhanced EasyAmplicon 2 by adding intergroup comparison, biomarker classification, and functional prediction modules (**Figure** **4**A–E). A genus‐level heatmap highlights distinct microbial community compositions across feces, plaque, and saliva (Figure 4A). A phylogenetic tree with evolutionary distances illustrates ASV distribution among groups (Figure 4B). Co‐occurrence networks constructed with ggClusterNet^[^ ^50^, ^51^ ^]^ reveal ASV interactions, with positive (purple) and negative (green) correlations (Figure 4C). Using published PacBio data,^[^ ^52^ ^]^ a random forest model identified microbial biomarkers, where bar plots show genus importance (colored by phylum) and an inset line plot guides optimal marker selection (Figure 4D). The pipeline integrates biomarker identification for direct use. Finally, functional annotation revealed significant intergroup differences in microbial functions. The heatmap indicates decreasing (blue) or increasing (red) trends, while the accompanying bar plots show the relative abundance of each function across groups (Figure 4E).

### 2.6 Customization and Interactive Reporting

For user‐friendly and reproducible reporting, the pipeline provides a customizable shell script file (StatPlot.sh) that allows users to tailor figure layouts, color palettes, taxonomic annotations, and statistical tests. It also supports the creation of publication‐ready multi‐panel figures, interactive HTML summaries, and structured tables for ecological or clinical insights.

### 2.7 Support for External Tools

While EasyAmplicon 2 offers a comprehensive solution for long‐read amplicon analysis, it also maintains compatibility with widely used third‐party microbiome analysis tools such as LEfSe,^[^ ^53^ ^]^ STAMP,^[^ ^48^ ^]^ and PICRUSt2.^[^ ^25^, ^54^ ^]^ Intermediate outputs (e.g., ASV tables, taxonomy profiles, and metadata files) are formatted to ensure seamless integration with these platforms, enabling researchers to extend and customize their analysis as needed.

### 2.8 Improvements of EasyAmplicon 2

EasyAmplicon 2 introduces several major improvements. First, the previous version did not include modules for third‐generation amplicon analysis, whereas in EasyAmplicon 2 we have added comprehensive support for third‐generation amplicon data, while further testing and optimizing workflows for short‐read amplicon analysis. Second, EasyAmplicon 2 provides user‐friendly pipelines tailored for mainstream long‐read datasets (Nanopore and PacBio), and integrates key software such as NanoFilt, Cutadapt, and Emu, supporting the complete analysis chain from quality control and denoising to taxonomic annotation. Third, we provide additional R scripts to facilitate the rapid generation of high‐quality figures for both short‐ and long‐read data. Meanwhile, the pipeline now supports multiple mainstream databases (e.g., GTDB and Emu) and includes optimized format conversion for different databases, improving the flexibility and compatibility of taxonomic annotation. In addition, the file structure on GitHub has been reorganized, with “Nanopore” and “PacBio” folders for long‐read data analysis and result output, and the introduction of a Snakemake‐based automated workflow (the “Snakemake” folder). Finally, the user guide has been revised (https://github.com/YongxinLiu/EasyAmplicon/wiki), now covering multiple installation options for software dependencies, a quick‐start guide, pipelines for both short‐ and long‐read amplicon data, as well as statistical and visualization options.

EasyAmplicon 2 addresses key challenges in long‐read amplicon data analysis through several notable innovations. It provides dual‐platform support for both PacBio and ONT sequencing technologies, employing advanced denoising algorithms to achieve species‐level resolution from single‐pass reads. The pipeline integrates taxonomic classification with ecological and functional interpretation in a seamless analytical workflow. In addition, it automates the generation of more than 30 publication‐ready visualizations through an intuitive interface that removes coding barriers. EasyAmplicon 2 also incorporates built‐in modules for biomarker discovery and network analysis within a fully reproducible R framework, offering researchers a comprehensive and user‐friendly solution for microbial community analysis.

Using mock datasets, we found that the short‐read, PacBio, and ONT amplicon analysis pipelines in EasyAmplicon 2 all demonstrated high accuracy in microbial annotation and quantification at the genus level. Based on the average of three replicates, the short‐read, PacBio, and ONT pipelines recovered 92.00%, 99.07%, and 94.20% of the theoretical relative abundance at the genus level, respectively (Figure S3A, Supporting Information). However, the short‐read pipeline provided substantially less species‐level taxonomic resolution (52.74% of the theoretical relative abundance across replicates), whereas the PacBio (95.07%) and ONT (85.39%) pipelines achieved much higher species‐level accuracy (Figure S3B, Supporting Information). When benchmarked against other existing third‐generation amplicon analysis pipelines using the same mock data, EasyAmplicon 2 (96.27% and 85.39% of the theoretical relative abundance across replicates for PacBio and ONT, respectively) outperformed all alternatives for both PacBio (23.94% for ampliseq, and 30.30% for Hifi‐16S‐workflow) and ONT (23.31% for NanoCLUST, and 81.80% for TRANA) datasets (Figure S3C,D, Supporting Information). In addition, the relative abundance of incorrectly annotated species (i.e., species not present in the mock community) was higher in other pipelines compared with EasyAmplicon 2. For PacBio mock data, the error rates were 41.03% for ampliseq and 16.06% for Hifi‐16S‐workflow, compared with only 3.73% in EasyAmplicon 2 (Figure S3C, Supporting Information). For Nanopore mock data, the error rates were 56.04% for NanoCLUST and 18.19% for TRANA, versus 14.61% in EasyAmplicon 2 (Figure S3D, Supporting Information). Overall, the third‐generation pipelines implemented in EasyAmplicon 2 deliver superior accuracy in microbial species annotation.

## 3 Discussion

The rapid evolution of long‐read sequencing technologies, particularly those developed by PacBio and ONT platforms, has revolutionized microbial ecology by enabling full‐length amplicon sequencing.^[^ ^55^, ^56^, ^57^, ^58^ ^]^ Unlike traditional short‐read approaches that target only partial gene regions, these technologies enable the recovery of complete 16S rRNA genes and other marker regions, thereby providing unprecedented taxonomic resolution at the species level.^[^ ^57^, ^59^ ^]^ However, the adoption of long‐read amplicon sequencing has been limited by several computational challenges, including relatively higher sequencing error rates (5–15%),^[^ ^60^ ^]^ incompatibility with established short‐read pipelines,^[^ ^61^ ^]^ and the lack of standardized analytical frameworks.^[^ ^62^ ^]^

To address these challenges, we developed EasyAmplicon 2, a modular and user‐friendly pipeline specifically tailored for long‐read amplicon analysis. Building upon the success of its predecessor,^[^ ^19^ ^]^ EasyAmplicon 2 integrates recent algorithmic advances in error correction^[^ ^63^ ^]^ and taxonomic classification,^[^ ^64^ ^]^ along with enhanced visualization capabilities. The pipeline uniquely bridges the gap between short‐ and long‐read workflows, while maintaining compatibility with existing microbiome analysis tools or databases.^[^ ^25^, ^65^ ^]^

Third‐generation amplicon sequencing (PacBio/ONT) offers key advantages of full‐length 16S/ITS coverage, enabling higher genus‐ and species‐level resolution while reducing misclassification associated with short‐read fragments.^[^ ^35^, ^66^ ^]^ However, its higher raw error rates and typically lower sequencing depth may hinder the detection of low‐abundance taxa, making computational workflows (e.g., circular consensus sequencing combined with DADA2) essential for error correction.^[^ ^67^ ^]^ Although several studies have demonstrated superior species‐level annotation with long‐read platforms,^[^ ^68^ ^]^ short‐read sequencing remains advantageous for stable abundance estimation and the detection of rare taxa. Differences in genus‐level abundances between third‐generation and short‐read annotation pipelines likely arise from variations in read length and accuracy, primer bias, database coverage, taxonomic classification methods, and downstream normalization or filtering.^[^ ^35^, ^69^ ^]^ In this study, using mock data and identical annotation methods, we also observed discrepancies in genus‐level abundances between short‐ and long‐read sequences, which may reflect differences in read length, sequencing accuracy, or primer bias. We recommend third‐generation sequencing for microbial amplicon studies, as it provides markedly improved species‐level resolution. EasyAmplicon 2 further confirms that the use of long‐read amplicon data significantly enhances species‐level annotation rates. By integrating the latest databases and software, our pipeline achieves superior annotation accuracy.

To save time, the pipeline provides two convenient options for software download, one from the Nature Microbiology Data Center and the other via Baidu Netdisk. We have also updated the software and R packages required for long‐read sequencing data analysis on Baidu Netdisk. To enhance the efficiency of analyzing and interpreting long‐read amplicon data, EasyAmplicon 2 includes detailed instructions and step‐by‐step explanations. Its streamlined installation and user‐friendly design significantly improve the overall efficiency of long‐read amplicon sequencing analysis. The pipeline is computationally efficient, allowing it to run on standard laptops while remaining compatible with high‐performance computing environments. This accessibility, along with markdown‐based reporting^[^ ^70^ ^]^ and standardized outputs compatible with QIIME 2,^[^ ^65^ ^]^ LEfSe,^[^ ^71^ ^]^ and STAMP,^[^ ^48^ ^]^ makes EasyAmplicon 2 particularly valuable for promoting reproducible research.

As long‐read sequencing gains increasing attention and application, a number of pipelines and online platforms have been developed for processing such data, including TRANA (https://github.com/genomic‐medicine‐sweden/TRANA), HiFi‐16S‐workflow (https://github.com/PacificBiosciences/HiFi‐16S‐workflow), ampliseq,^[^ ^72^ ^]^ NanoCLUST,^[^ ^73^ ^]^ NanaRTax,^[^ ^74^ ^]^ SituSeq,^[^ ^75^ ^]^ PRONAME,^[^ ^46^ ^]^ and “long‐read‐tools.”^[^ ^76^ ^]^ However, many of these existing pipelines or platforms are not specifically designed for long‐read amplicon sequencing data, or have not been thoroughly tested and optimized, or tend to produce installation errors that are difficult to resolve. To address these limitations, EasyAmplicon 2 integrates widely used methods for analyzing long‐read amplicon data generated by PacBio and Nanopore platforms and validates its annotation accuracy. In this study, comparative analysis demonstrated that EasyAmplicon 2 achieves higher accuracy in microbial species annotation than other evaluated pipelines. This advantage likely stems from its integration of the latest software and databases, providing a more accurate and user‐friendly solution for amplicon data analysis. Moreover, unlike other pipelines that focus exclusively on either PacBio or Nanopore data, EasyAmplicon 2 offers a comprehensive framework encompassing both types of third‐generation amplicon data, along with versatile visualization tools. While EasyAmplicon 2 delivers superior species‐level annotation, the comparison results may be influenced by the choice of tools and software, and should be interpreted with caution. Overall, our findings highlight EasyAmplicon 2 as a robust and versatile pipeline for microbial amplicon analysis.

Although the comparison results demonstrate the advantages of EasyAmplicon 2 in amplicon data analysis, several aspects remain open for future improvement. Future development will focus on three key areas: 1) benchmarking existing long‐read amplicon analysis software to further optimize the pipeline; 2) enhancing computational efficiency for processing ultra‐deep sequencing datasets (> 10 million reads); and 3) expanding functionality by broadening support for diverse marker genes and adding more analysis and visualization options for long‐read amplicon data. Additionally, cloud‐based deployment options are under development to facilitate large‐scale collaborative studies, and an online analysis platform supporting both short‐ and long‐read amplicon data is also in future plans for users without coding experience, further enhancing accessibility.

## 4 Conclusion

In summary, EasyAmplicon 2 provides a powerful, comprehensive, and easy‐to‐use solution for analyzing full‐length amplicon data generated from long‐read sequencing platforms. This pipeline has the characteristics of high operating efficiency and covers multi‐platform (Illumina, PacBio, or Nanopore) data by offering an integrated pipeline for quality control, denoising, taxonomic annotation, diversity analysis, biomarker detection, and functional prediction. By supporting major classification databases and offering seamless visualization options, EasyAmplicon 2 empowers researchers to uncover detailed ecological patterns and functional mechanisms in microbial communities. As full‐length sequencing continues to gain traction in microbial ecology, clinical microbiomics, agriculture, and environmental science, EasyAmplicon 2 emerges as an essential solution for high‐resolution amplicon analysis, enabling deeper insights into the structure and function of complex microbiomes.

## 5 Experimental Section

### Pipeline Implementation and Architecture

EasyAmplicon 2 is built on a modular architecture that combines Shell scripting for core processing tasks with R for statistical analysis and visualization. This dual‐language design ensures computational efficiency for large‐scale data processing while providing flexibility for advanced statistical analysis. The pipeline supports two modes of operation: 1) a command‐line interface for batch processing of large datasets, and 2) an interactive RStudio interface for exploratory analysis and visualization. In addition, we implemented an automated version of EasyAmplicon 2 using the Snakemake workflow management system,^[^ ^77^ ^]^ enabling task scheduling, dependency tracking, and parallel execution, which further enhances portability and reproducibility. Cross‐platform compatibility has been validated on Windows (via Git Bash), macOS, and Linux systems.

Data Sources and Sequencing Platforms

The sequencing data used in this study were obtained from previously published amplicon sequencing projects targeting three types of human microbiome samples (gut, subgingival plaque biofilm, and saliva)^[^ ^15^ ^]^ as well as environmental samples.^[^ ^78^ ^]^ These datasets span three major sequencing platforms including Illumina, PacBio, and ONT.

The Illumina and PacBio datasets were both derived from the same human sample types with periodontitis (an oral inflammatory disease) of stage III and IV grades A‐B, enabling a direct comparison of different sequencing strategies. Illumina data were generated on the MiSeq platform by amplifying the V3‐V4 region of the 16S rRNA gene, producing standard paired‐end short reads. In contrast, PacBio data were obtained using the Sequel II platform with circular consensus sequencing (CCS) technology, generating high‐accuracy, full‐length 16S rRNA sequences. This dual‐platform design facilitates an in‐depth evaluation of microbial diversity resolution across sequencing technologies.

ONT data were sourced from an environmental microbiome study that included four sampling locations (South, North, East, and Outside). Library preparation and sequencing were performed using the Oxford Nanopore 16S Barcoding Kit (SQK‐16S024), Flow Cell Priming Kit (EXP‐FLP002), and MinION R9 flow cells (FLO‐MIN106D, Oxford Nanopore Technologies, UK). Basecalling and demultiplexing were conducted using the GPU‐accelerated Guppy software (version 6.4.6, Oxford Nanopore Technologies). Reads were quality‐ and length‐filtered to retain sequences between 1300 and 1650 bp with an average Q‐score of at least 10. Corresponding environmental parameters were also collected to support downstream ecological and statistical analyses.

Data Processing

Raw sequencing data in FASTQ format (from either PacBio, Oxford Nanopore, or Illumina platforms) underwent an optimized quality control process incorporating read merging (for paired‐end data), quality filtering (Q‐score ≥20), and adapter removal using modified parameters specific to each sequencing technology. For long‐read data, a hybrid error‐correction approach was implemented by combining CCS for PacBio HiFi reads^[^ ^8^ ^]^ and adaptive alignment with signal‐level correction for Nanopore data.^[^ ^60^ ^]^ Sequence dereplication and chimera removal were performed based on the SILVA 138.2 database.^[^ ^28^ ^]^ The denoising process employs an optimized DADA2^[^ ^61^ ^]^ algorithm with platform‐specific parameterization, while a traditional 97% similarity clustering method remains available for OTU‐based analysis.

Taxonomic and Phylogenetic Analysis

Taxonomic annotation of 16S rRNA gene sequences was carried out using three alternative approaches: 1) the SINTAX algorithm (implemented in VSEARCH) for rapid reference‐based classification;^[^ ^21^ ^]^ 2) DADA2 for denoising and generating ASVs,^[^ ^35^ ^]^ followed by taxonomy assignment using reference databases; and 3) the Emu default database with its built‐in classifier,^[^ ^11^ ^]^ with database formats adapted for compatibility across workflows. These three methods could be applied independently or in combination, depending on analytical requirements. Phylogenetic reconstruction was performed using the maximum likelihood (ML) method, with bootstrap support used to assess branch reliability. Phylogenetic reconstruction is conducted using maximum likelihood estimation supported by bootstrap values.^[^ ^79^ ^]^

Validation Analysis

To compare EasyAmplicon 2 with version 1.0 and evaluate the accuracy of our pipeline, the workflow was validated using mock samples. The simulated mock datasets were obtained from a previously published study.^[^ ^18^ ^]^ The short‐read amplicon pipeline and the PacBio pipeline in EasyAmplicon 2 were applied, both utilizing the Emu default database^[^ ^11^ ^]^ to perform annotation comparisons on the mock data. To further demonstrate the performance of EasyAmplicon 2, it was also benchmarked with existing third‐generation amplicon analysis pipelines, including TRANA, Hifi‐16S‐workflow, ampliseq,^[^ ^72^ ^]^ and NanoCLUST,^[^ ^73^ ^]^ and compared annotation accuracy across all pipelines that could be successfully executed. For details on the use of mock data and comparison, please refer to the description in the Supporting Information.

Statistical Analysis

All data were analyzed using R software (v 4.3). The analytical framework includes comprehensive diversity assessments, with α‐diversity metrics such as Shannon,^[^ ^80^ ^]^ Simpson,^[^ ^81^ ^]^ Chao1,^[^ ^82^ ^]^ and β‐diversity measures including weighted/unweighted UniFrac^[^ ^83^ ^]^ and Bray–Curtis dissimilarity.^[^ ^84^ ^]^ Differences in α‐diversity between groups were assessed using the two‐sided Wilcoxon rank‐sum test, while PERMANOVA (permutational multivariate analysis of variance) was applied to test for significant differences in β‐diversity measures. Differential abundance analysis was performed using linear discriminant effect size analysis (LEfSe).^[^ ^71^ ^]^ Specifically, the non‐parametric Kruskal–Wallis rank‐sum test was first applied to detect species with significant differences in abundance between groups. For species identified as significant, the Wilcoxon rank‐sum test was further used to examine consistency among subgroups. Subsequently, linear discriminant analysis (LDA) was conducted to evaluate the effect size (LDA score) of each feature. Biomarker identification was also performed using random forest classification.^[^ ^85^ ^]^ Co‐occurrence network analysis was conducted with ggClusterNet 2,^[^ ^51^ ^]^ while functional prediction was carried out using PICRUSt2.^[^ ^25^ ^]^ MaAsLin 2^[^ ^86^ ^]^ was used to test the difference of function abundance between groups. Visualization components were implemented with ggplot2,^[^ ^87^ ^]^ extended by phyloseq,^[^ ^88^ ^]^ and amplicon^[^ ^19^ ^]^ to produce standardized, publication‐ready figures in PDF format. The pipeline also generates interactive HTML reports with parameter tracking and customizable RMarkdown templates to ensure reproducibility of results. All quantitative data were presented as mean ± standard error of the mean (mean ± SEM). *p*‐value < 0.05 was considered statistically significant.

### Ethics Statement

No ethics approval was required for this study because all samples used were publicly available and obtained from open‐access sources.

## Conflict of Interest

The authors declare no conflict of interest.

## Author Contributions

H.L., D.B., Z.Z., S.Y., H.Y., J.X., and M.Z. contributed equally to this work. Y.‐X.L., R.G., T.W., T.C., X.C., X.L., and D.B. conceived and coordinated the study. H.L. and D.B. were responsible for the overall implementation planning of the pipeline code development. Z.Z. led the development of the Nanopore analysis pipeline and performed overall code testing. S.Y. and H.Y. led the development of the PacBio analysis pipeline and contributed to overall code testing. H.L., D.B., Z.Z., S.Y., and H.Y. prepared the initial draft of the manuscript. All other authors contributed to pipeline testing and manuscript revision. All authors reviewed and approved the final manuscript for publication.

## Supporting information



Supporting Information

## Data Availability

The pipeline source code, installation packages, and documentation are maintained at https://github.com/YongxinLiu/EasyAmplicon. Test datasets and benchmarking results are available under BioProject PRJNA933120 and PRJNA1045017.
