# Supplementary material for: EasyAmplicon 2: Expanding PacBio and Nanopore Long Amplicon Sequencing Analysis Pipeline for Microbiome
Source: Adv Sci (Weinh). 2025 Oct 27;13(1):e12447. doi: 10.1002/advs.202512447 (PMC12767004; doi:10.1002/advs.202512447)
Supplement: Supplementary file 1 — Supporting Information [file ADVS-13-e12447-s001.docx]

**SUPPORTING INFORMATION**

**Validation of EasyAmplicon 2 using mock data**

In the Methods section, we briefly describe how mock datasets were used to evaluate the accuracy of bacterial taxonomy annotation in EasyAmplicon 2. Specifically, we assessed the annotation accuracy of both short-read and long-read analysis pipelines using mock data from Illumina, PacBio, and Nanopore sequencing platforms. Each platform included three samples, with three replicates per sample. The data were annotated with the corresponding pipeline, and the average relative abundance at the genus or species level across replicates was calculated to generate the stacked bar plots. Incorporating replicates minimizes errors from individual samples and provides a more reliable representation of the true community composition. For clarity, we further calculated the relative abundance percentages of each annotated genus/species as reference indicators of pipeline accuracy. We counted the relative abundance percentage of the genera/species that were annotated to the genus/species but not in the mock data as “Other” and the genus/species without genus/species information as “Unclassified”. “Other” can be used as a simple indicator to reflect the relative proportion of pipeline annotation errors, and the combination of “Other” and “Unclassified” can be used as a simple indicator to reflect the accuracy of the pipeline annotation.

To further evaluate the accuracy of species-level annotation in EasyAmplicon 2, we compared its PacBio amplicon analysis module with other commonly used PacBio pipelines, including ampliseq (https://github.com/nf-core/ampliseq), and HiFi-16S-workflow (https://github.com/PacificBiosciences/HiFi-16S-workflow). Similarly, we compared the Nanopore amplicon analysis module in EasyAmplicon 2 with NanoCLUST,^[74]^ and TRANA (https://github.com/genomic-medicine-sweden/TRANA). Other third-generation amplicon pipelines were excluded from the comparison because they consistently produced errors that could not be resolved despite multiple attempts. For fair comparison, all pipelines were configured with the same annotation database (Emu default database).^[31]^


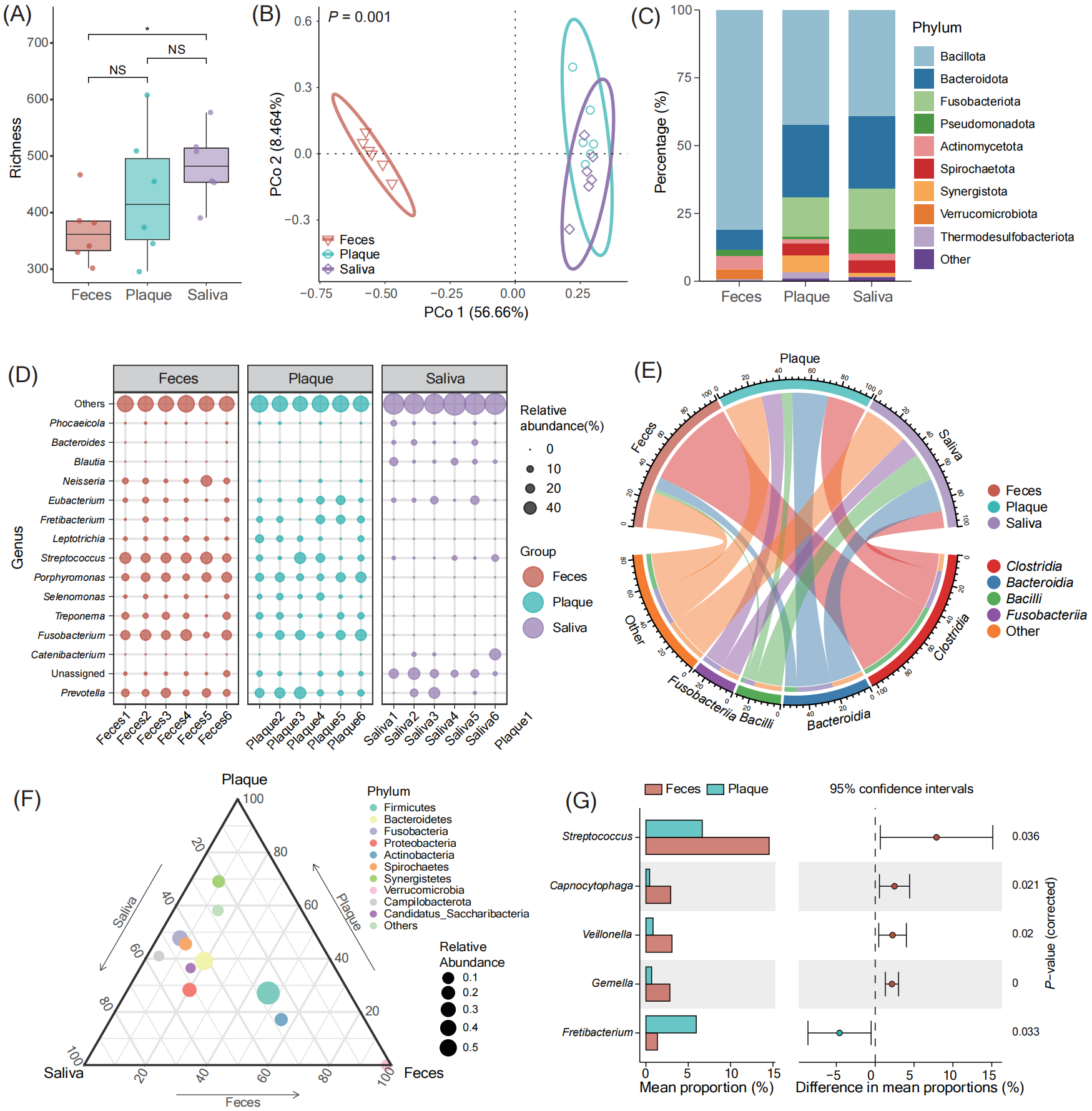
Figure S1 Examples of illustration based on short-read data using EasyAmplicon 2. (A) Box plot shows the richness difference among groups. (B) Principal coordinate analysis (PCoA) displays the beta diversity difference among groups. (C) Stacked bar plot shows the change of relative abundance of bacteria at phylum level. (D) Grouped bubble plot shows the variation in relative abundance of microbial genera between samples. (E) The chord diagram shows the relative abundance of the main genera in different groups. (F) Ternary plot showing the relative abundance of microbial phyla at three sampling sites. (G) The extended error bar plot shows the relative abundance differences of microbial genera between groups. (n = 6 samples per condition, the significance test method used in extended bar plot is two-sided Wilcoxon rank-sum test)


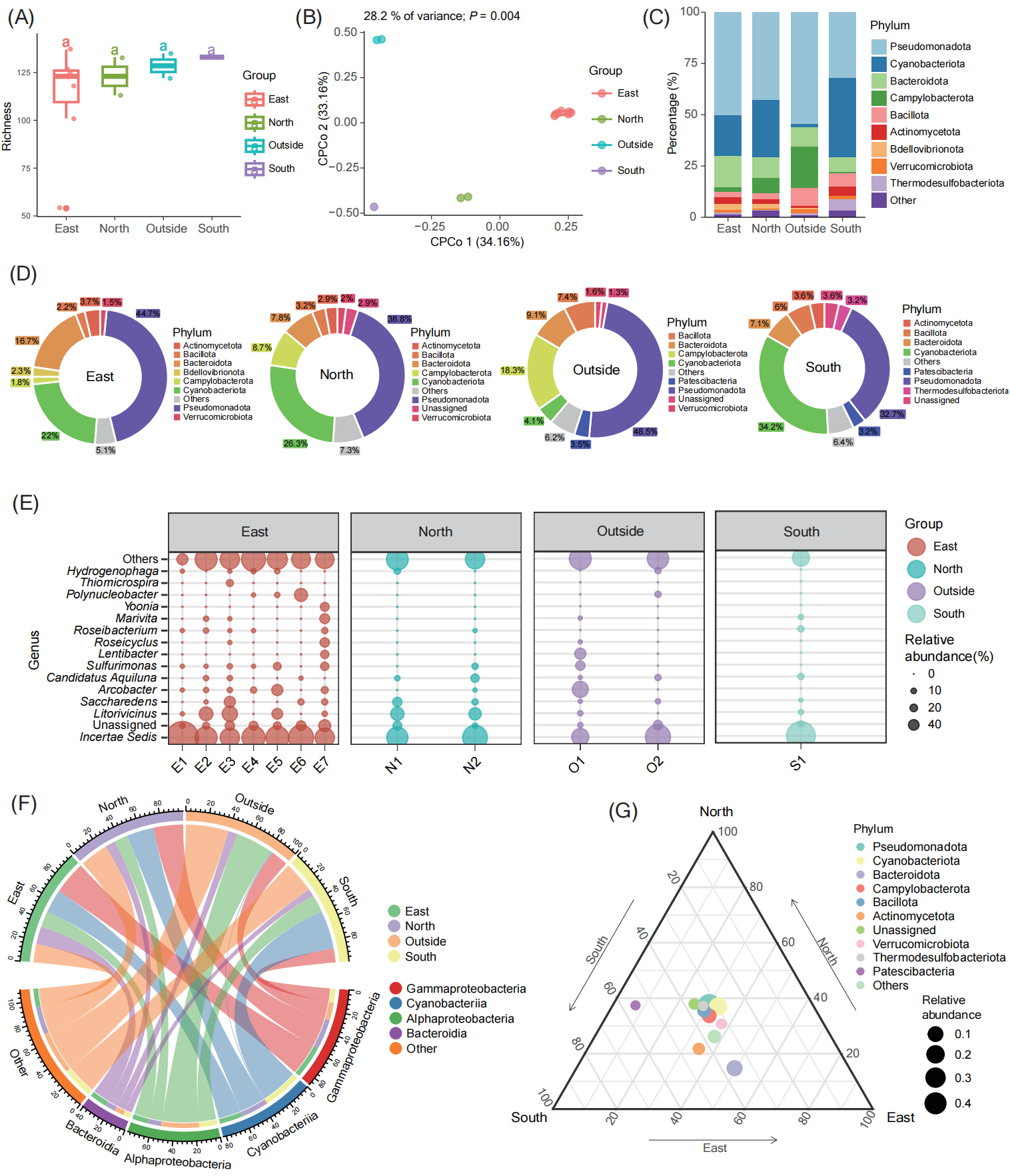
Figure S2 Examples of illustration based on Nanopore sequencing data using EasyAmplicon 2. (A) Box plot shows the richness difference among groups. (B) The constrained principal coordinate analysis (CPCoA) displays the beta diversity difference among groups using Bray-Curtis distance. (C) Stacked bar plot shows the change of relative abundance at bacteria phylum level among four groups. (D) The donut plot shows relative abundance at bacteria phylum level among four groups. (E) Grouped bubble plot shows the variation in relative abundance of microbial genera between samples. (F) The chord diagram shows the relative abundance of the main genera in different groups. (G) Ternary plot showing the relative abundance of microbial phyla at the four sampling sites. (n = 7 samples for East group; n = 2 samples for North group; n = 1 samples for South group; n = 2 samples for Outside group).


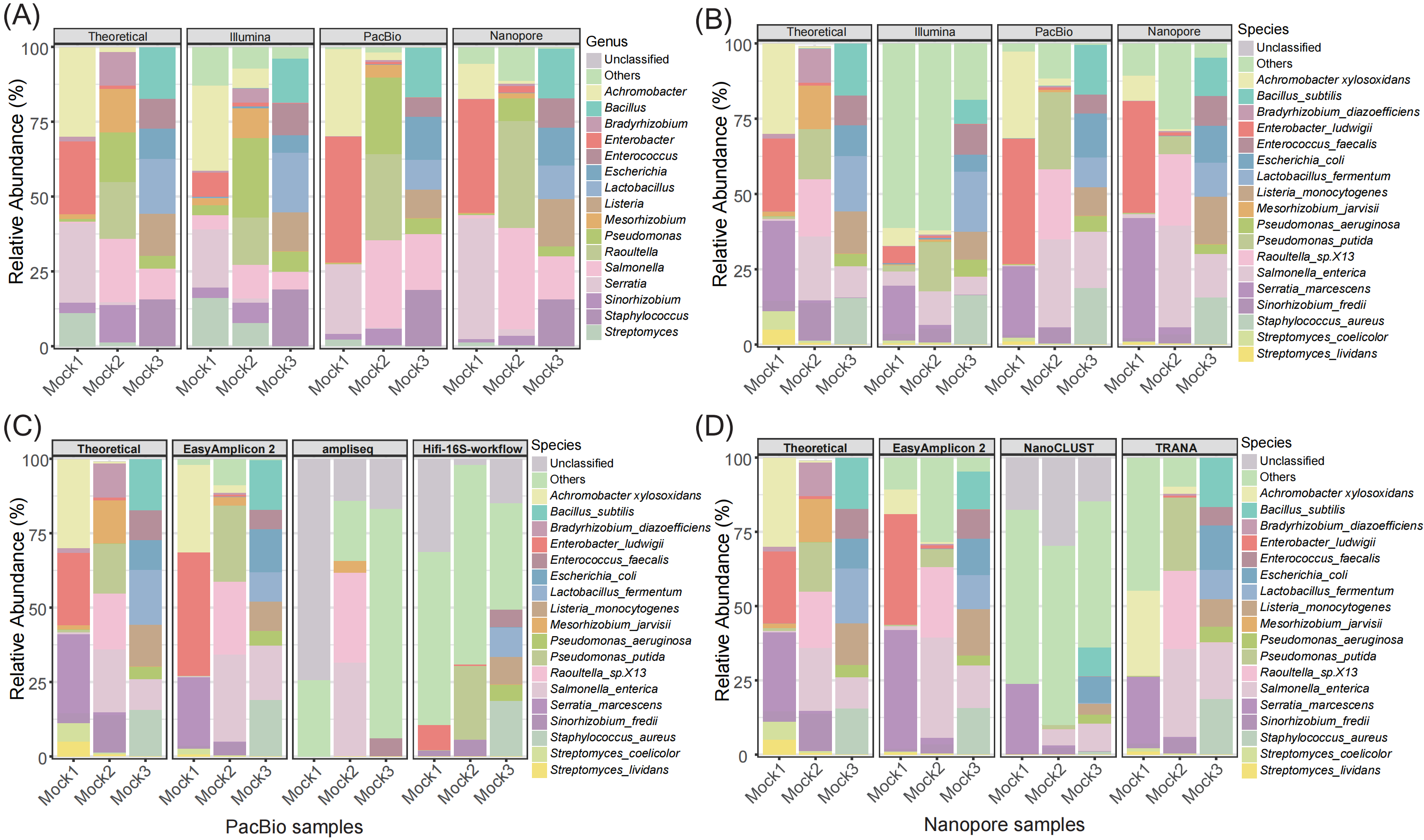
Figure S3 The validation analysis of performance of EasyAmplicon 2. Using a mock data, we analyzed the data using the short-read amplicon analysis pipeline, the PacBio analysis pipeline, and the Nanopore analysis pipeline included in EasyAmplicon 2, and compared the genus-level (A) and species-level (B) microbiome compositions obtained by different pipelines. Using the same mock data, we compared the species-level annotation results of the existing PacBio (C) and Nanopore (D) third-generation amplicon analysis pipelines with EasyAmplicon 2. (n = 3 replicate samples for Mock1, Mock2, and Mock3)
